# Mitochondrial genomes of soft scales (Hemiptera: Coccidae): features, structures and significance

**DOI:** 10.1186/s12864-023-09131-9

**Published:** 2023-01-21

**Authors:** Congcong Lu, Xiaolei Huang, Jun Deng

**Affiliations:** State Key Laboratory of Ecological Pest Control for Fujian and Taiwan Crops, College of Plant Protection, Fujian Agriculture and Forestry University, Fuzhou, 350002 China

**Keywords:** Coccidae, Mitogenome comparison, Codon usage bias, Positive selection

## Abstract

**Background:**

Soft scales (Hemiptera: Coccidae), including important agricultural and forestry pests, are difficult to identify directly by morphological characters. Mitochondrial genomes (mitogenomes) have been widely used in species identification and phylogenetic research. However, only three complete mitogenomes, and very few mitochondrial genes of scale insects (Hemiptera: Coccoidea) can be searched in GenBank. Mitogenome comparisons between scale insects or between scale insects and other hemipteran species have not yet been reported.

**Results:**

In this study, detailed annotation of three new mitogenomes and comparative analysis of scale insects were completed, as well as comparative analysis of the gene composition, gene arrangement, codon usage and evolutionary forces between scale insects and 488 other hemipteran species for the first time. We found that high A + T content, gene rearrangement and truncated tRNAs are common phenomena in soft scales. The average A + T content and codon usage bias of scale insects are higher and stronger than those of other hemipteran insects, respectively. The *atp8* gene of Hemiptera and nine other protein-coding genes of scale insects are under positive selection with higher evolutionary rates.

**Conclusions:**

The study revealed the particularity of the scale insect mitogenomes, which will provide a good reference for future research on insect phylogenetic relationships, insect pest control, biogeography and identification.

**Supplementary Information:**

The online version contains supplementary material available at 10.1186/s12864-023-09131-9.

## Background

Soft scales (Hemiptera: Coccidae) are an economically important group with small body sizes and piercing-sucking mouthparts [1, 2]. They include notorious agricultural pests such as *Ceroplastes rubens* and *C. floridensis*, which have been known for centuries, not only for severe damage to crops and plants but also for the value of wax industrial products from *Ericerus pela* [2]. There are 1223 species in Coccidae [3], which have high biodiversity and great variation in morphology among different families than other Sternorrhyncha species [4, 5]. General swelling of the body and sclerotization of the dorsum in adulthood of soft scales frequently make identification difficult with only morphological characteristics [1, 6, 7]. In the last two decades, genetic data from the mitochondrial genome (mitogenome) have been widely used in different insect groups for species identification and analyses of phylogenetics, biogeography and molecular evolution due to scarce recombination and stable gene composition [8, 9]. However, when searching all sequences from mitochondrial genes of soft scales in GenBank, the results showed that there were only 241 *cox1*, 8 *cox2*, and zero other mitochondrial sequences from 241 species (accessed on May 17, 2022). A lack of mitochondrial gene sequences has severely hindered the development of coccidology involving identification, phylogenetics, biogeography, etc.

With the development of sequencing technology, the number of mitogenomes available from different orders is increasing rapidly [10]. The number of complete mitogenomes of Hemiptera reached 1486 sequences in GenBank (accessed on May 17, 2022). However, the high A + T content (> 84%) with AT dinucleotide repeats makes it more difficult to obtain complete mitogenomes of scale insects (Hemiptera: Coccoidea) [11–14]. Two parasitized wasp mitogenomes investigated in a recent study [13] were mistaken for scale insect host mitogenome sequences [15]. To date, only three Coccidae mitogenomes (*Ceroplastes japonicus* [12], *Saissetia coffeae* [11] and *Didesmococcus koreanus* [14]) have been made available in GenBank. Our team published the first two complete mitogenomes of Coccidae in 2019 and 2020. The novel structures and features in these two mitogenomes lead us to believe that the work of Coccidae mitogenomes is not over yet. More mitogenomes of soft scales are needed to understand the unique evolutionary pattern of this group. Systematic differences in the A + T content of insect mitochondrial genes indicate that cellular metabolism has changed during evolution [16]. Mutation bias in the evolution process can occur with a selective advantage or a selective mutation [17]. High A + T content might be related to codon usage and evolutionary forces of mitogenomes of soft scales. However, studies on the codon usage and evolutionary pressures of scale insect mitogenomes are missing. For the three known Coccidae mitogenomes, approximately half of the mitochondrial tRNAs are truncated tRNAs with a lost T-arm or D-arm [11, 13, 14], and the gene arrangements are also different from those of other hemipteran mitogenomes [11–14], especially tRNA genes. Are these characteristics common in Coccidae mitogenomes? Systematized comparative analysis of the mitochondrial genomes between soft scales and other hemipteran insects needs to be performed.

Mitochondria are important places for energy production, and all mitochondrial protein coding genes (PCGs) play an integral role in metabolism adjustment during environmental adaptation [18]. For functional requirements, most mitochondrial PCGs experienced stronger purifying selection in strongly locomotive species, and insects, birds, and mammals with weak locomotive ability have higher evolutionary rates than those with strong locomotive ability [19, 20]. Mutation bias and natural selection (purifying selection, neutrality and positive selection) are important evolutionary forces that have been revealed by nucleotide composition bias, transcription and the number of accumulated beneficial or fixed deleterious mutations [20, 21]. These factors can be combined in codon usage pattern detection to determine and compare the extent of their effects [22]. All amino acids in insect mitogenomes are encoded by two or more codons, which are used unevenly and show different patterns in different genes and species for adapting to environmental pressures [23, 24]. The preferential use of a synonymous codon to encode amino acids in different genes is called codon usage bias, which is the result of the balance between mutation bias and selection pressures [24, 25]. Describing the codon usage patterns will help us to explore the underlying processes and long term molecular evolution.

In the present study, we sequenced and annotated three new complete mitogenomes of Coccidae (*Ceroplastes rubens*, *Ceroplastes floridensis*, and *Ericerus pela*). *Ceroplastes japonicas* sequenced by Deng et al. (2019) was reannotated due to some tRNA loss in the initial annotation [12]. A comparative analysis of the sequence and genome structure of five Coccidae mitogenomes from three different genera and all other hemipteran species was reported based on nucleotide composition, codon usage and evolutionary forces. Furthermore, phylogenetic trees inferred from concatenated nucleotide sequences and amino acid sequences of 13 mitogenome PCGs were analysed to reconstruct the relationships within hemipteran insects.

## Results

### Genome base composition of five scale insects

**Fig. 1:**
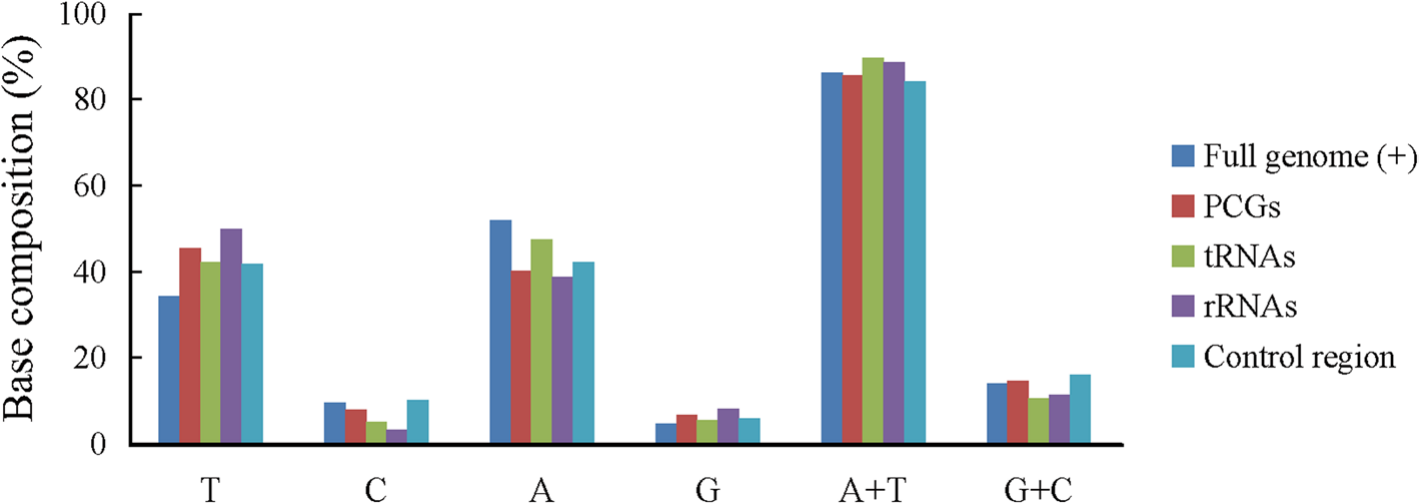
Average base composition of different organization of five scale insect mitogenomes are represented by different colours (navy blue: the complete mitogenomes, red: protein coding genes (PCGs), green: transfer RNAs (tRNAs), purple: ribosome RNAs (rRNAs), indigo: control region)

The mitogenome lengths of *C. japonicus*, *C. rubens*, *C. floridensis* and *E. pela* were 14,977 bp, 15,316 bp, 15,085 bp and 16,349 bp, respectively (annotation information is presented in Tables S1, S2, S3 and S4). The 37 typical genes of the four mitogenomes have all been annotated (Fig. S1). The sizes of the PCGs, tRNAs and rRNAs of these mitogenomes were similar, but the size of the CR varied greatly. Overlapping regions and intergenic spacer regions with different sizes were also found. The base composition and mitogenome organization of five scale insects (including *S. coffeae*) are shown in Fig. 1 and Table S5. The average A + T content of the five complete mitogenomes was 86.1%. Additionally, the A + T content of *E. pela* was 88.4%, which is the highest among insects at present. The A + T content of tRNAs of five scale insects was 89.6%, which was higher than that of other types of genes. Approximately half of the tRNAs in each mitogenome were truncated, without a T arm/loop or DHU arm/loop (Figs. S2, S3, S4 and S5). The gene order of the four scale insects was consistent with that of *S. coffeae* (Lu et al. 2020) but different from the proposed ancestral gene order (*Drosophila yakuba*) [26].

### Genome structure and composition of scale insects and other hemipteran mitogenomes

**Fig. 2:**
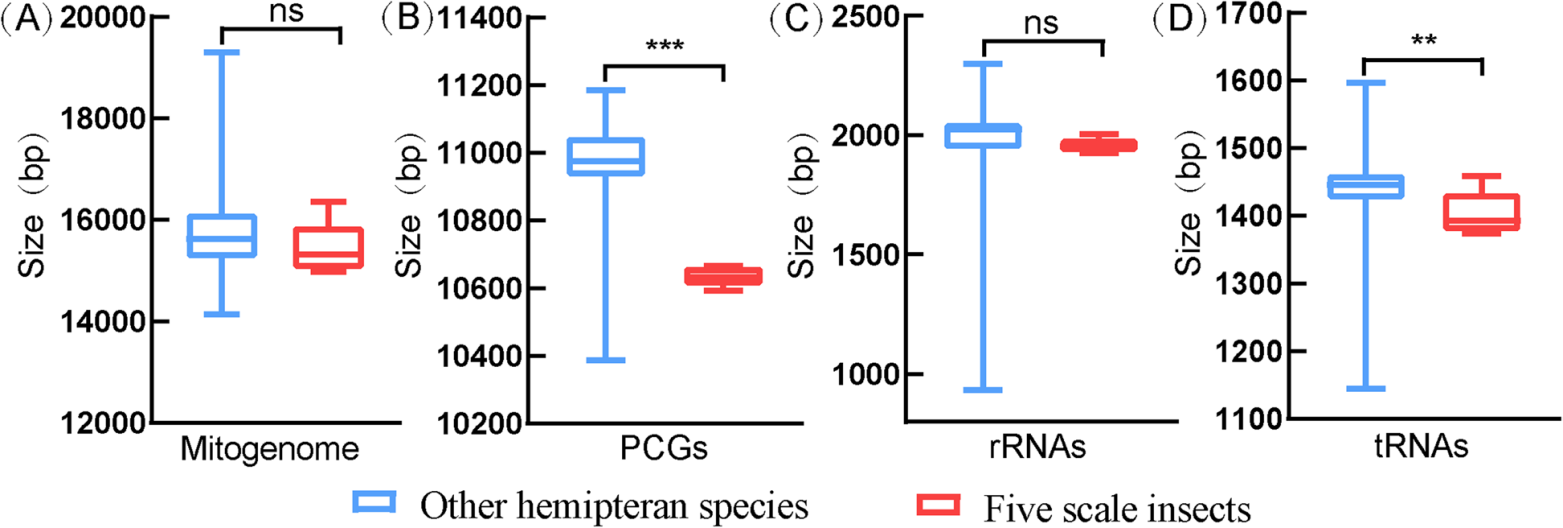
Box plots of the size of the complete mitogenome and PCGs, rRNAs, and tRNAs of five scale insects and other hemipteran insects. Boxes represent the first and third quartiles, and the horizontal line represents the median. The asterisks (***), (**) and ns above the bars indicate highly significant differences (*p* < 0.01), significant differences (*p* < 0.05) and no significant difference between the two groups, respectively

**Fig. 3:**
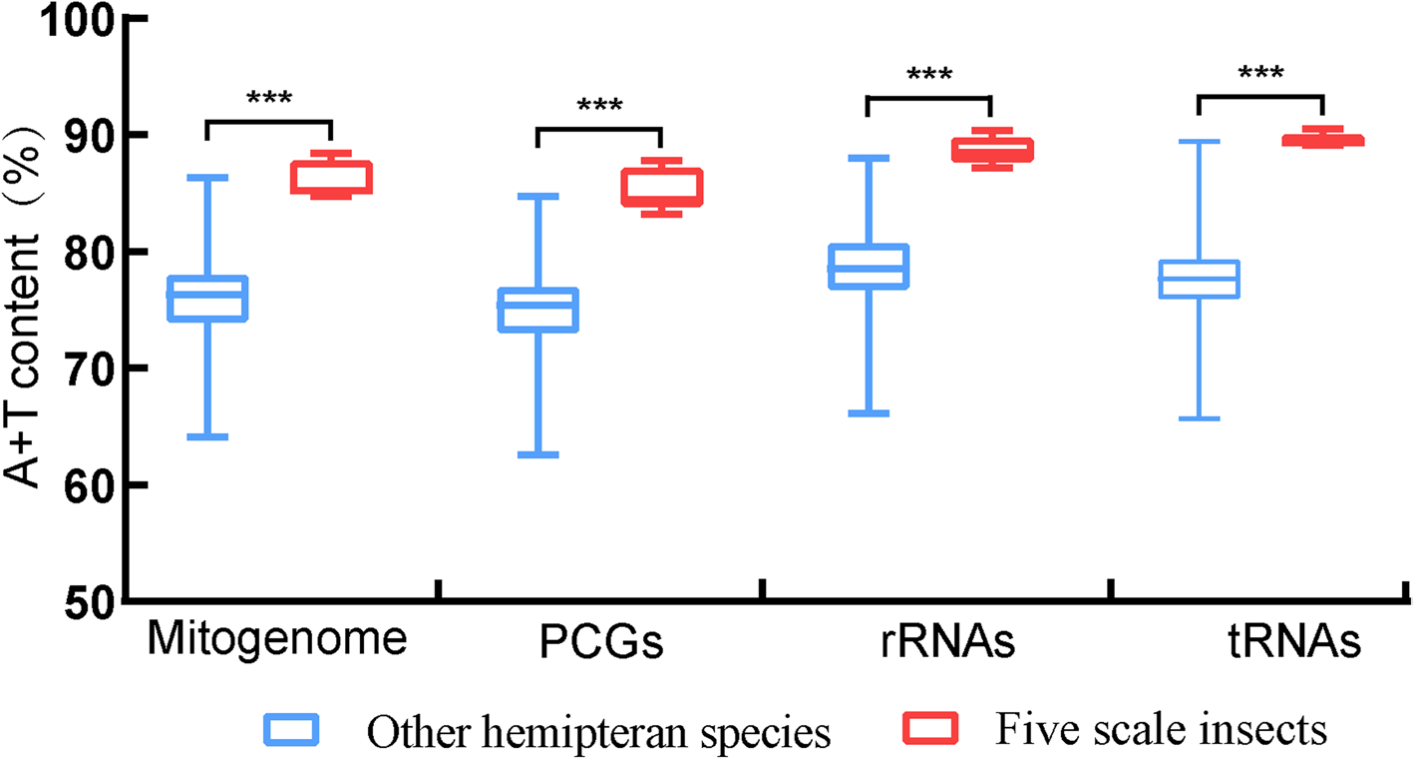
Box plots of the A + T content of complete mitogenome PCGs, rRNAs, and tRNAs of five scale insects and other hemipteran insects. Boxes represent the first and third quartiles, and the horizontal line represents the median. The asterisks (***) above bars indicate that there is a highly significant difference (*p* < 0.01) between the two groups

**Fig. 4:**
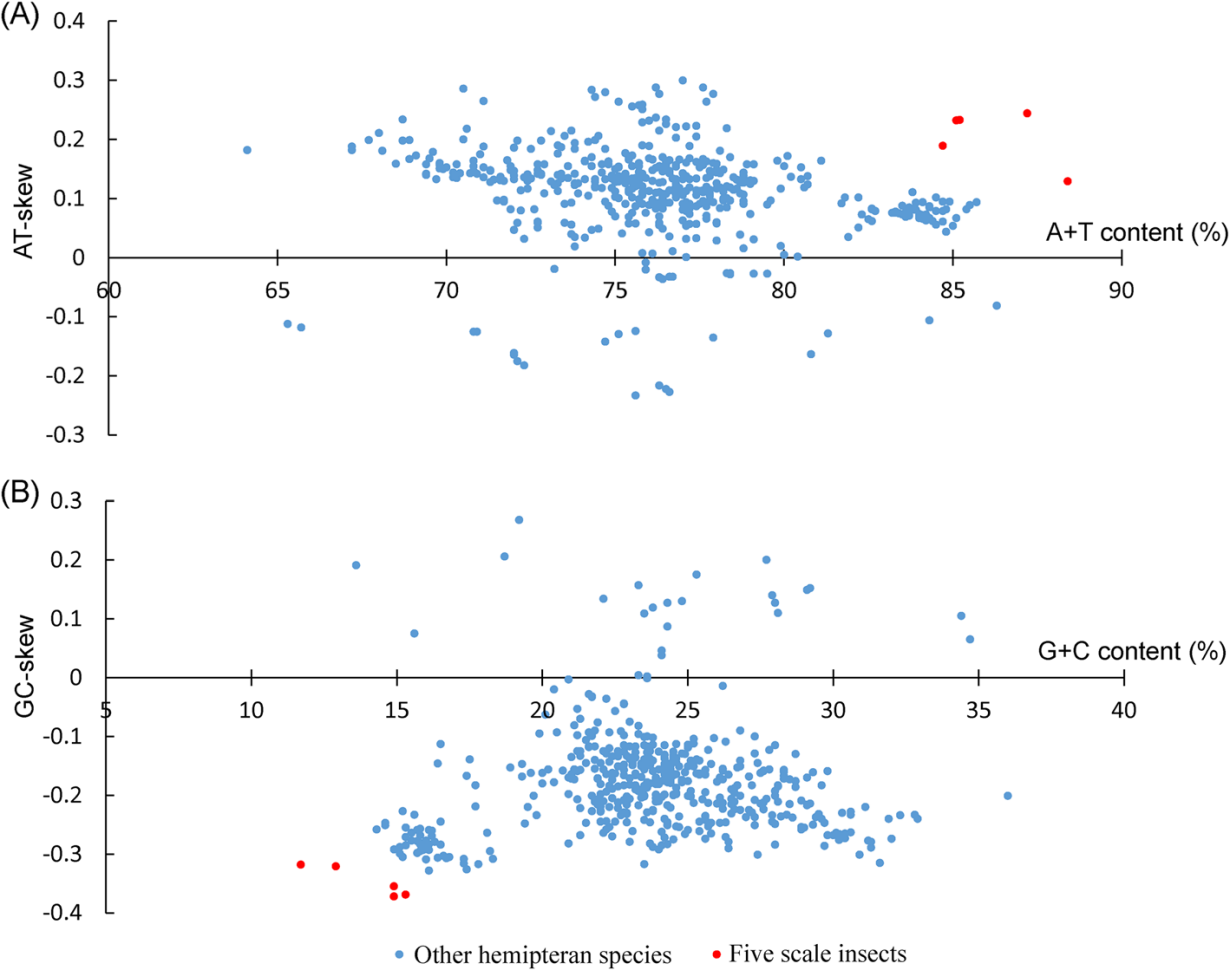
Nucleotide composition across five scale insect and other hemipteran insect complete mitogenomes: (A) A + T content and AT skew; (B) G + C content and GC skew

The size of scale insect mitogenomes fluctuated from 14,977 bp to 16,349 bp, with an average size of 15,423 bp. The size of other hemipteran mitogenomes fluctuated from 14,143 bp to 19,295 bp, with an average size of 15,723 bp. The sizes of the mitogenomes, PCGs, rRNAs and tRNAs are shown in Fig. 2. The sizes of mitogenomes and rRNAs were similar, but the sizes of PCGs and tRNAs of scale insects were significantly smaller than those of other hemipteran species. The average A + T contents of scale insect mitogenomes, PCGs, rRNAs and tRNAs were significantly higher than those of other hemipteran species (Fig. 3). According to our analysis, the A + T content of other hemipteran mitogenomes ranged from 64.1% (*Aradus compar*) to 86.3% (*Aleurodicus Dugesii*), with an average of 76.3%. However, the mitogenomes of the five scale insects showed a strong bias of A/T bases, with an average A + T content of 86.1%, which was significantly higher than that of other hemipteran species. The AT-skew and GC-skew are shown in Fig. 4. For scale insects and most other hemipterans, A and C bases were used more frequently than T and G bases, and the A + T content was significantly higher than the G + C content. In other hemipteran species, incomplete stop codons T or TA were common in the *cox1*, *cox2* and *nad5* genes, which were not found in the scale insect mitogenome.

### Analysis of relative synonymous codon usage

**Fig. 5:**
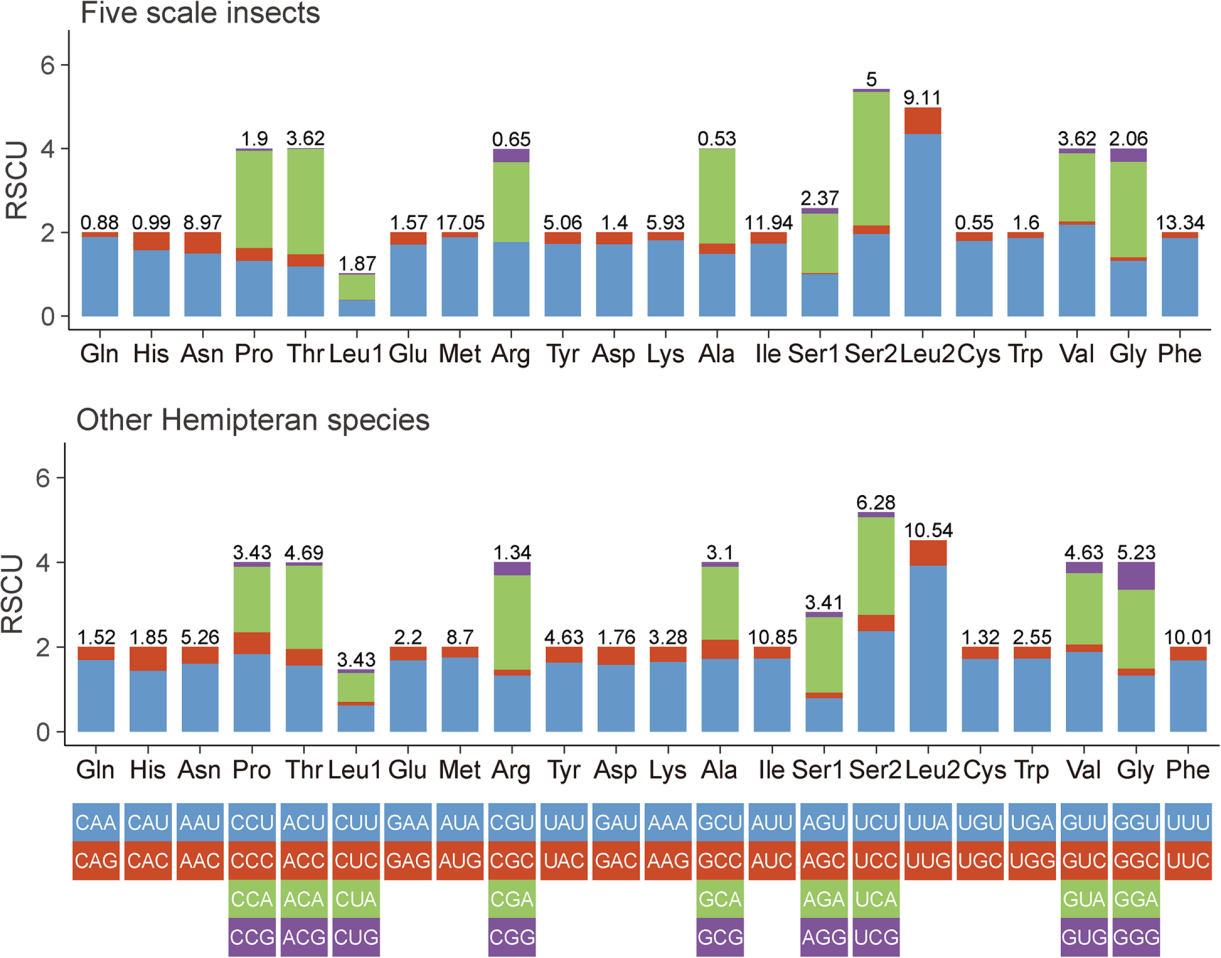
Relative synonymous codon usage (RSCU) in the mitogenomes of five scale insects and other hemipteran species. Codon families are labelled on the x-axis. Values on the top of the bars denote amino acid usage. Stop codons are not given

The RSCU results revealed that 62 codons (excluding 2 stop codons) were used unequally (Fig. 5), and the third position of frequently used codons was mostly A or U. In particular, AUU was the most frequent codon for isoleucine (Ile) in hemipteran species (Fig. 5 and Table S6), while UUU was the most frequent codon for phenylalanine (Phe) in five scale insects (Fig. 5 and Table S7). Approximately half of codons (RSCU > 1) were preferred in five scale insects and other hemipteran species. Four codons (UUU-Phe, UUA-Leu2, AUU-Ile and AUA-Met) were consistently used as frequent codons in five scale insects and other hemipteran species that were only composed of A or U. In other hemipteran species, there were 22 codons (RSCU > 1.6, mean RSCU: 1.92) that were overrepresented and 29 codons (RSCU < 0.6, mean RSCU: 0.26) that were underrepresented. However, in five scale insects, there were 21 codons (RSCU > 1.6, mean RSCU: 2.12) that were overrepresented, 29 codons (RSCU < 0.6, mean RSCU: 0.18) that were underrepresented, and two codons (GCG-Ala and CGC-Arg) without A or U that were not even used. The codon usage pattern of the five scale insects was not significantly different from that of other hemipteran species, but the preference for codon usage was stronger in the five scale insects.

### Analysis of the effective number of codons (ENC)

**Fig. 6:**
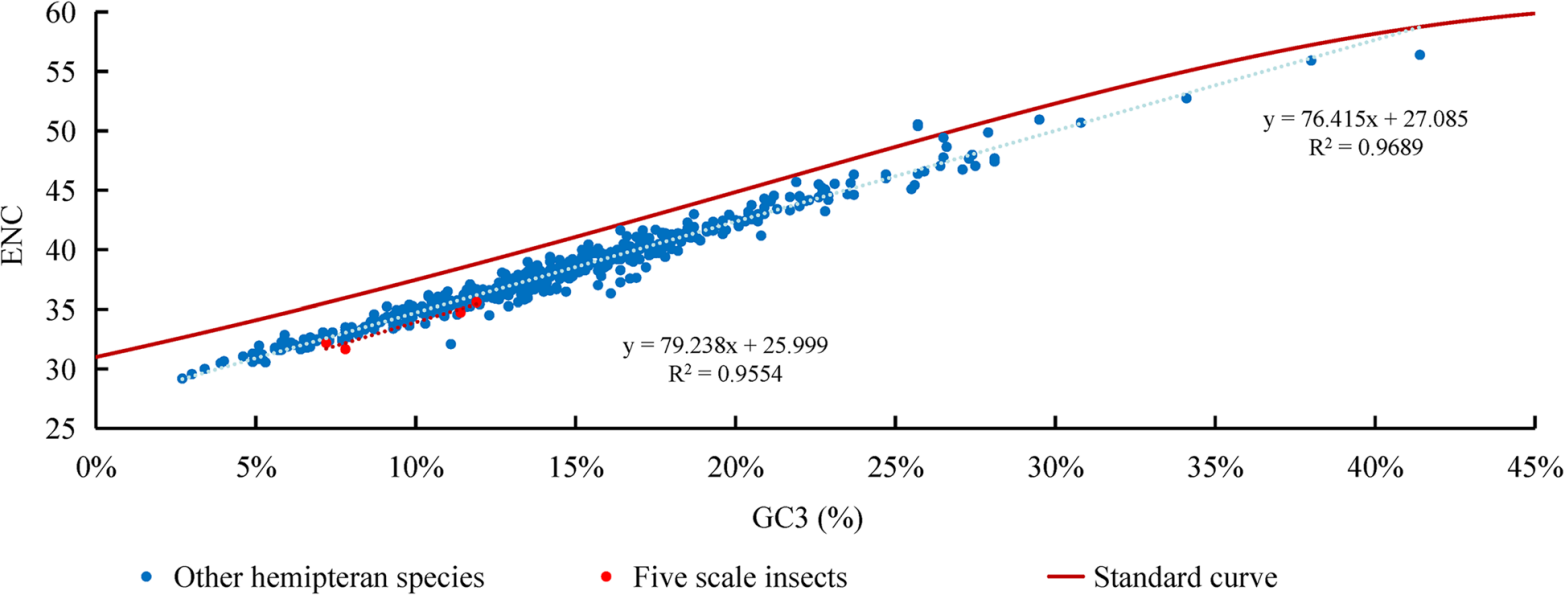
ENC-GC3 plot of PCGs in the mitogenomes of five scale insects and other hemipteran species. The standard curve describes the relationship between the ENC and average GC content in the third positions of PCG codons (GC3) without selection pressure

The ENC values of five scale insects varied from 31.67 to 35.61 (with an average value of 33.95), and those of other hemipteran species varied from 29.19 to 56.39 (with an average value of 38.00) (Fig. 6). The average value suggested a generally strong codon usage bias in Hemiptera. The ENC values of the five scale insects were significantly lower (*p* < 0.05) than those of the other hemipteran species. In five scale insects, fewer types of codons were used to produce proteins, indicating that their mitogenomes were more likely to undergo stronger selection pressure. The strong correlation between ENC and GC3 of five scale insects (F = 64.22, *p* < 0.01) and other hemipteran species (F = 15,171.78, *p* < 0.01) indicated that the G/C composition of the 3rd position of codons was positively related to ENC and negatively related to codon usage bias, but codon usage patterns were not notably different between the two groups. Except for two species of Aleyrodidae, all other species were distributed below the standard ENC curve (Fig. 6), indicating the presence of natural selection and suggesting that codon usage patterns were not only caused by mutations.

### Neutrality plot analysis

**Fig. 7:**
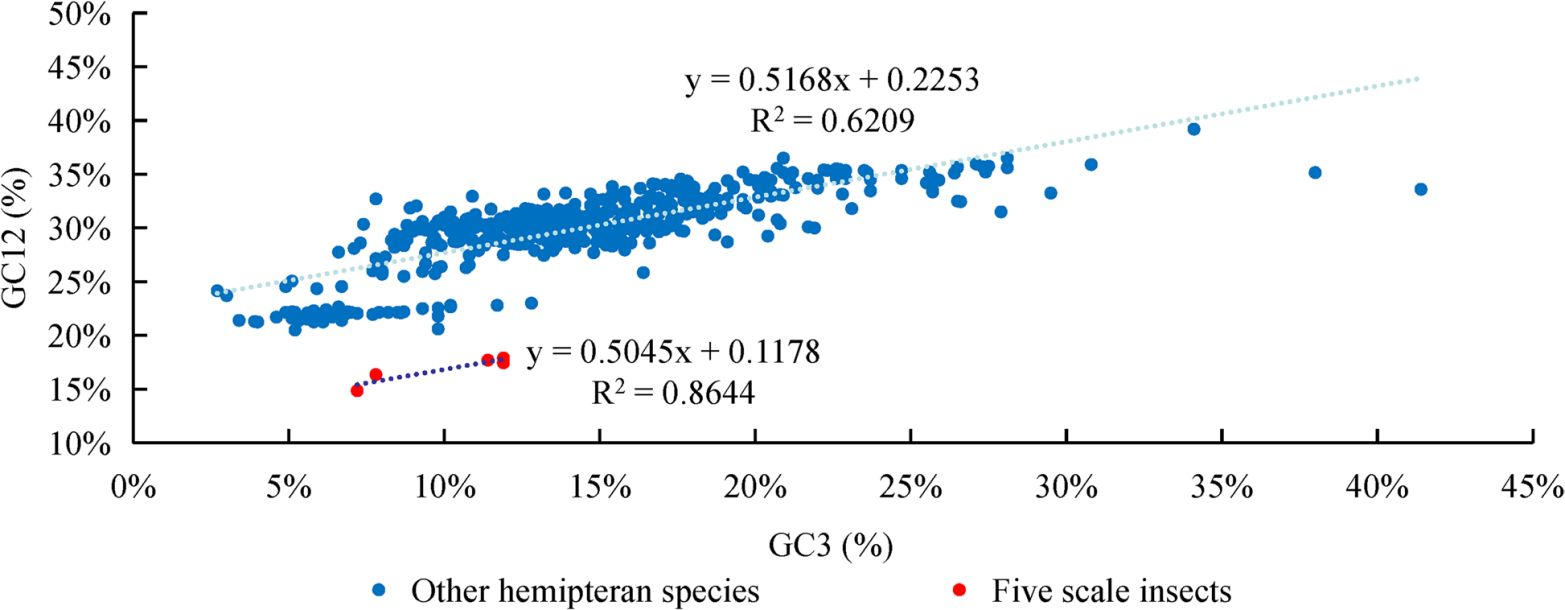
Neutrality plot analysis of GC12 and GC3 content of PCGs in the mitogenomes of five scale insects and other hemipteran species. Regression analysis was conducted with the average GC content in the first and second positions (GC12) against that in the third position (GC3) of the PCG codons of the mitogenome

The neutrality plot results showed that there was a significant correlation between GC12 and GC3 in five scale insects (F = 19.13, *p* < 0.05) and most hemipteran species (F = 797.49, *p* < 0.01) (Fig. 7), indicating that mutation pressure directly affected all codon positions. The dots were distributed with a wide range of the GC3 distribution, which suggested that mutation pressure has an effect on codon usage bias for Hemiptera. The slopes of the regression line of five scale insects and other hemipteran species indicated that mutation pressure (50.45% for scale insects and 51.68% for other hemipteran species) and natural selection (49.55% for scale insects and 48.32% for other hemipteran species) both influenced codon usage. Taken together, these results suggest that mutation pressure acting on all codon positions played a more important role than natural selection in determining codon usage bias.

### Evolutionary rate

**Fig. 8:**
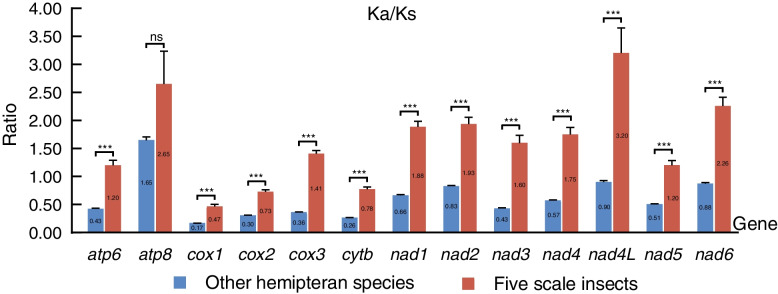
Comparisons of average Ka/Ks ratios for 13 protein-coding genes in other hemipteran insects and five scale insect complete mitogenomes. The asterisk (***) and ns above the bars indicate highly significant differences (*p* < 0.05) and no significant difference between the two groups, respectively

The Ka/Ks ratio ranged from 0.47 (*cox1*) to 3.20 (*nad4L*) in five scale insects and from 0.17 (*cox1*) to 1.65 (*atp8*) in other hemipteran insects (Fig. 8). Except for the *atp8* gene, the Ka/Ks ratios of PCGs in five scale insects were all significantly higher than those in other hemipteran species. In other hemipteran species, in addition to the *atp8* gene (Ka/Ks = 1.65), all the Ka/Ks ratios of PCGs were less than 1, indicating that these genes were under purifying selection but that *atp8* was under positive selection with the highest evolutionary rate. However, in scale insects, except for the Ka/Ks values of *cox1*, *cox2* and *cytb,* which were below 1 and were under purifying selection with relatively slow rates, the values of other PCGs were all greater than 1, indicating that these genes were under intense positive selection. As the function of positive selection is to preserve beneficial mutations and purifying selection is to rapidly remove deleterious mutations, the comparisons of average Ka/Ks ratios revealed that adaptive evolution is likely to strongly affect codon usage of amino acids in five scale insects but only slightly in other hemipteran species.

### Gene rearrangement

**Fig. 9:**
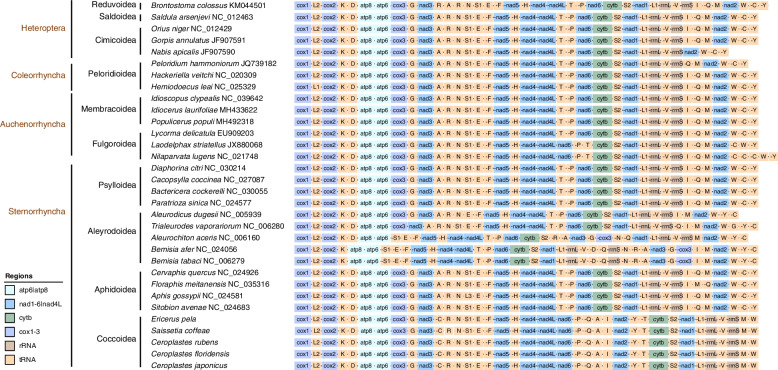
The gene arrangement of 32 representative mitogenomes of Hemiptera. Genes with negative signs are located on the minor (N) strand, and other genes are located on the major (J) strand. Different gene types are shown with different colours (light blue: *atp6*/*atp8*, blue: *nad1*–*6*/*nad4L*, green: *cytb*, purple: *cox1*–*3*, brown: *rRNA*, orange: *tRNA*)

To clearly show the gene order of five scale insects and other hemipteran miotgenomes, 27 representative species of Hemiptera were selected for comparison with five scale insects. The taxonomic status and gene order of each species are shown in Fig. 9. The four gene clusters of all species (*cox1*-*trnL2*-*cox2*-*trnK*, *atp8*-*atp6*, *nad5*-*trnH*-*nad4*-*nad4L* and *nad1*-*trnL1*-*rrnL*-*trnV*-*rrnS*) were relatively conserved. The gene orders of some families in Coleorrhyncha, Auchenorrhyncha and Sternorrhyncha were relatively conserved. However, the mitogenomes of Aleyrodoidea in Sternorrhyncha were highly rearranged and involved PCGs and tRNA genes. For other groups, the rearrangement mainly occurred among tRNA genes. The gene order of scale insects was significantly different from that of other Hemiptera, but the gene rearrangement mainly occurred among tRNA genes, and the gene block between *nad4L* and *trnW* was the hot spot of rearrangement.

### Phylogenetic analysis

**Fig. 10:**
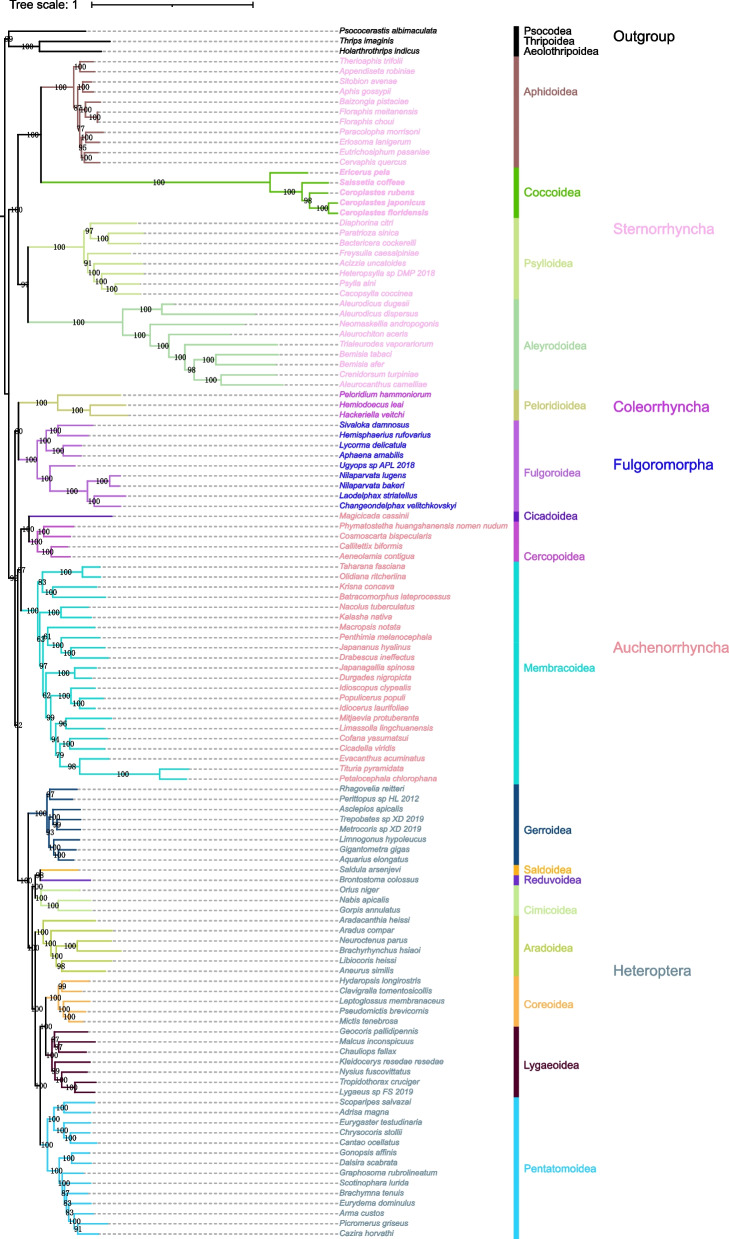
The maximum likelihood tree is constructed from the nucleotide sequences of 13 mitochondrial PCGs of the five scale insects and 114 other hemipteran species using IQ-TREE. The ultrafast bootstrap values are shown at the nodes. The species from the same suborder/infraorder are shown with the same colours, and the branches from the same subfamily are also shown with the same colours

The maximum likelihood trees showed stable topological structures with high nodal support values (Fig. 10), which was very similar to previous phylogenetic trees based on hemipteran mitogenomes [27, 28]. Sternorrhyncha was placed as sister to all other Hemiptera in the phylogenetic tree and emerged near the base of the tree. The five scale insects were clustered into a single clade in Sternorrhyncha with 100% bootstrap values, recovering Coccoidea as the sister taxon of Aphidoidea. The species of Aphidoidea, Psylloidea and Aleyrodoidea in Sternorrhyncha were all clustered into single clades with high nodal support values. Interestingly, the branch representing scale insects was especially prominent in the phylogenetic trees, which was related to the high evolutionary rates of mitochondrial PCGs.

## Discussion

### High A + T content in scale insects

Nucleotide bias is a common phenomenon in most insect mitogenomes, with unequal use of four bases always presenting high A + T content [10, 29]. The A + T content of holometabolous insects is usually high, and that of Hymenoptera is higher than that of other species [30]. Additionally, Sternorrhyncha was the group with the highest A + T content (80.97%) in Hemiptera [31]. The possible explanation that mitogenomes tend to evolve with high A + T content may be that guanine (G) and cytosine (C) nucleotides require more energy than adenine (A) and thymine (T) nucleotides for biosynthesis and are not as readily available in cells [32]. Cytosine deamination to uracil is a common spontaneous biochemical change in DNA that elevates G:C → A:T mutation rates [33]. Scale insects might show an evolutionary adaptation to host plants that are deficient in organic nitrogen [34, 35]. The elevated mitochondrial substitution rate in Hymenoptera may be related to the parasitic lifestyle, which has been tested by comparing parasitic and nonparasitic species [36]. Meanwhile, infection with the endosymbiont *Wolbachia* may increase the substitution rate of *Drosophila recens* mitogenomes, as proposed by Shoemaker et al. in 2004 [37]. Widespread *Ophiocordyceps*-allied fungus was found to be the dominant endosymbiont of Coccidae species, which is vertically transmitted from mother to offspring [38, 39]. The possible relationship between the endosymbionts and mitochondrial DNA composition of Coccidae species needs to be confirmed in the future.

### Condon usage pattern

Incomplete stop codons TA or T were common in other hemipteran mitogenomes, which may be illustrated by posttranscriptional modification in that these incomplete codons will be added “A” to the 3′-end of mRNA through polyadenylation to form a complete stop codon TAA to terminate transcription [40]. Codons coding for the same amino acid are not equally used in protein products, either among different organisms or among genes from a single species [41]. In the mitogenomes of five scale insects and other hemipteran species, codons ending with A/U bases were used more frequently, while codons ending with C/G bases were avoided. Furthermore, codon usage patterns indicated by the RSCU and ENC values seem to show different trends between scale insects and other hemipteran species. The codon usage bias in scale insects is stronger than that in other hemipteran species. Codon usage patterns are often influenced by selective pressure and mutation pressure [25]. This may be due to the high A + T content sequences in mitogenomes leading to high codon bias. The ENC and neutrality plots are used to detect the putative proportion of evolutionary forces [42, 43]. In the present study, we found that mutation pressure played a slightly more important role in codon usage bias, and selection pressure also played a potential role in gene expression level and amino acid composition [44]. While the exact cause of this phenomenon is still unknown, there is no doubt that there is a balance between natural selection (gene length, gene function, and translational selection) and mutational bias (base content, mutational position) [25].

### Selection pressures

Molecular evolution of the mitogenome is driven by strong purifying selection, which has been documented in some papers [18, 45, 46]. The protein products of mitochondrial genes are vital for the mitochondrial oxidative phosphorylation process; thus, they are more restricted in their function [22, 47]. However, evidence of positive selection on the insect and vertebrate mitochondrial gene by NI estimation has been reported [48]. Recently, there has also been some evidence for significant positive selection of mitochondrial PCGs in insects, such as mosquitoes [49], grasshoppers [50] and neopterans [51], for energy demand and environmental adaptation. An analysis of the evolutionary rates of the mitochondrial PCGs of 90 hemipteran species in 2015 showed that all genes underwent purifying selection (Ka/Ks < 1), with the *atp8* gene showing the highest evolutionary rate and the *cox1* gene showing the lowest [52]. Our results for other hemipteran insects (488 species) were consistent with these results, but the *atp8* gene was different and restricted by positive selection (Ka/Ks = 1.65). Our study containing more data can more precisely show the evolutionary forces of hemipteran insects. However, nine PCGs have higher nonsynonymous mutation rates (Ka/Ks > 1) in scale insect mitogenomes, and the dominant evolutionary force seems to be positive selection, which rapidly preserves the adaptive changes in amino acids.

The 13 PCGs of mitogenomes encode subunits in the electron transport chain where ATP, carbon dioxide and water are generated by oxidization of carbon-hydrates and fats [47]. In addition, the metabolic power for locomotion has a linear correlation with speed [53]. Shen et al. (2010) suggested that the important genes in the oxidative phosphorylation pathway underwent adaptive evolution in bats, which may be the result of increased metabolic requirements related to the evolution of flight [54]. Weakly flying and flightless birds accumulate more nonsynonymous mutations relative to synonymous mutations in the mitogenomes, and the Ka/Ks ratio is negatively correlated with locomotive speed. Interestingly, more mitochondrial genes with positive selection are also detected in insects with weaker flight abilities than in those with stronger flight ability [51]. Most scale insects of Coccidae live on their host plants without moving because their legs gradually degenerate during development and they feed on the poor nutrients from the phloem sap of host plants [35]. Thus, we speculate that scale insects with weak or inactive locomotion might require less energy than other hemipteran species, which leads to adaptive evolution with their host organism and promotes the accumulation of more nonsynonymous mutations in mitogenome PCGs.

## Conclusion

The high A + T content and extraordinary structure may be the reasons why it is difficult to sequence, assemble and annotate the mitogenomes of scale insects. Except for the conserved genes (*cox1*, *cox2* and *cytb*) with stable functions, which are often used as marker genes, the other 10 PCGs are all affected by positive selection, indicating a high evolutionary rates of scale insect mitogenomes. Three newly sequenced mitogenomes of scale insects in this study will improve the current understanding of mitogenome structure and phylogenetic research of Coccoidea and will provide a reference for future mitogenome assembly. More mitogenome data and new methods are needed to investigate the specific evolutionary process of Coccidae.

## Methods

### DNA extraction and species identification

The collected information is shown in Table 1. The specimens were preserved in 95% ethanol and stored in a − 80 °C freezer. Total genomic DNA of each specimen was extracted with a DNeasy Blood & Tissue Kit (Qiagen, Hilden, Germany) following the manufacturer’s instructions. The quality of the extracted DNA was assessed through 0.8% agarose gel electrophoresis and then stored at − 20 °C for future use. The *cox1* sequences were amplified with the primers C1-1554F and C1-2342R from Deng et al. [6]. Based on morphological descriptions and molecular identification, three specimens were identified as *Ceroplastes rubens*, *Ceroplastes floridensis* and *Ericerus pela*.

**Table 1:** List of the scale insects in the present study

Sample ID	Place	Host plant	Species name	Reference
S2017–175	Fuzhou, Fujian Province	*Ixora chinensis*	*Saissetia coffeae*	(Lu et al. 2020)
S2017–230	Ningbo, Zhejiang Province	*Gardenia jasminoides*	*Ceroplastes japonicus*	(Deng et al. 2019)
S2017–670	Kunming, Yunnan Province	*Fatsia japonica*	*Ceroplastes rubens*	OP388828
S2018–52	Ningbo, Zhejiang Province	*Ligustrum quihoui*	*Ericerus pela*	OP388829
S2020–4	Fuzhou, Fujian Province	*Camphora officinarum*	*Ceroplastes floridensis*	OP388830

### Sequence assembly and gene annotation

The DNA of *Ceroplastes rubens* was sent to Sangon Biotech Company (Shanghai, China), and *Ericerus pela* and *Ceroplastes floridensis* were sent to Novogene Bioinformatics Technology Co., Ltd. (Tianjin, China) for next-generation sequencing. At Novogene Co. Ltd., the Illumina TruSeqTM DNA Sample Prep Kit (Illumina, San Diego, CA, USA) was used to construct a library with an average insert size of 400 bp. Genomic DNA was sequenced on the HiSeq X Ten platform (2 × 150 bp paired-ended reads), and average sequencing depth was approximately 13x, 40x, 37x for *C. rubens*, *E. pela*, *C. floridensis,* respectively. After filtering, clean reads (4 G, 13 G, 12 G for *C. rubens*, *E. pela*, *C. floridensis,* respectively) with high quality were used for subsequent genome assembly. The mitogenomes were assembled with NOVOPlasty v 3.7.1 [55], MEGAHIT v 1.0 [56] and GetOrganelle v 1.7.1 [57]. The circular mitogenome sequences (15,316 bp, 16,349 bp, 15,085 bp) were corrected by Pilon [58]. Finally, the complete mitogenomes of three species were obtained. We also downloaded the mitogenome sequence of *Ceroplastes japonicus* for gene annotation. The preliminary annotation was completed using the annotation workflow of the scale insect mitogenome [11]. Some genes that were not detected by Mitos Web Server (MITOS2) [59] and ARWEN v 1.2 [60] were determined by alignment with closely related species. Therefore, the mitochondrial tRNAs of *Saissetia coffeae* were used as a reference to search for “lost” genes, and sequence alignment in gap regions between identified genes was based on the anticodon sequences. RNAplot in the ViennaRNA Package [61] was used to draw the secondary structure of tRNAs. The long noncoding region was defined as the control region. The online software CGview [62] was used to visualize the gene elements.

### Mitogenome comparative analysis

The mitogenomes of all 488 hemipteran species (excluding Coccidae) were downloaded from NCBI. The online software Interactive Tree Of Life (iTOL) v 4 [63] was used to visualize the gene order of all Hemiptera, and different genes were represented by different colours. Mitogenome rearrangement events were reconstructed by the CREx (Common Interval Rearrangement Explorer) online tool [64], with *Drosophila yakuba* [26] as a reference. The base composition (for the majority strand), AT/GC skew (for the majority strand), amino acid usage, and relative synonymous codon usage (RSCU) of each mitogenome were calculated using PhyloSuite v1.2.1 [65]. RSCU < 1 indicates that codons are used less frequently than the expected number, which is the total number of codons divided by the number of synonymous codons of each amino acid, while RSCU > 1 indicates that codons are used more frequently than expected [66]. Codons with RSCU > 1.6 are considered overrepresented codons, and codons with RSCU < 0.6 are considered underrepresented codons [67].

The ENC plotting analysis of ENC (y-axis) and GC3 (x-axis) was constructed to analyse the extent of codon usage bias and test if mutations are the only factor causing the codon usage pattern. The standard curve reflects the theoretical ENC values that are only caused by mutations, which is based on the equation ENC = 2 + s + {29/[s^2^ + (1 - s^2^)]}, where s means GC3, from Wright [43]. When the ENC value lies below the standard curve, it indicates the presence of selective effects. ENC values vary from 20, which means that only one codon is used for each amino acid (extreme codon usage bias), to 61, which means that all codons are equally used to code amino acids (no codon usage bias). The extent of codon usage bias increases as ENC approaches 20, and ENC < 35 is conventionally used to express strong usage. The neutrality plot analysis comparing GC12 (y-axis) and GC3 (x-axis) was constructed to explore the effect of mutation pressure and natural selection on the composition bias of Hemiptera. If the dots are distributed along the diagonal with a regression coefficient value approaching 1, it indicates that mutations play a more important role in codon usage bias. When dots are scattered and distributed with the regression coefficient value approaching zero, natural selection is more important [42]. The effective number of codons (ENC), the frequency of nucleotides G + C at the third codon positions (GC3) and the mean of the frequency of both G + C at the first and second position (GC12) of PCGs were calculated by Codon W 1.4 [68].

For exploring the evolutionary rates on the mitogenome PCGs of five scale insects and other hemipteran species, the average rate of nonsynonymous substitutions (Ka), the average rate of synonymous substitutions (Ks), and the average ratio of Ka/Ks of each PCG were calculated with DnaSP V 5.0 [69], based on *Drosophila yakuba* [26] (Diptera: Drosophilidae) as a reference. Ka/Ks < 1 indicates purifying or negative selection, Ka/Ks = 1 indicates neutrality, and Ka/Ks > 1 indicates positive selection. The ratio is closer to 1, suggesting that the selection pressure is smaller [22].

### Phylogenetic analysis

The topological structure of the phylogenetic tree may be affected by the high A + T content or poorly conserved PCGs of the scale insect mitogenome. Therefore, based on previous studies [27, 28, 70], a total of 119 representative species (including 3 species in this study) from 4 representative suborders of Hemiptera were selected in this study, and *Holarthrothrips indicus*, *Thrips imagines*, and *Psococerastis albimaculata* were selected as outgroups (see Table S8). The multiple alignments of each PCG sequence were conducted using MAFFT [71] and concatenated by PhyloSuite [65]. The conserved nucleotide sequences were selected by trimAL using the “-strictplus” command [72]. Substitution saturation was detected in DAMBE with Xia’s test [73, 74]. The results showed that the index for measuring substitution saturation was significantly lower than the critical value of the index, indicating that the datasets could be used to construct phylogenetic trees. The best-fit substitution models (GTR + F + R8) of nucleotide sequences were tested by ModelFinder [75]. For maximum likelihood (ML) analysis, phylogenetic trees were constructed by IQ-TREE v 1.6.12 [76], with 5000 replicates.

## Supplementary Information

**Additional file 1:** **Table S1.** Information of the species used in the phylogenetic analysis in this study.

**Additional file 2: Table S2.** Annotation and gene organization of the mitochondrial genome of *C. japonicus*. N: minor strand; J: major strand. The tRNAs were annotated by different methods: MITOS2, ARWEN or aligning with the sequences of related species that were not annotated by software (ALIGN). Running ARWEN with the “-ps < num>” option.

**Additional file 3: Table S3.** Annotation and gene organization of the mitochondrial genome of *C. rubens*. N: minor strand; J: major strand. The tRNAs were annotated by different methods: MITOS2, ARWEN or aligning with the sequences of related species that were not annotated by software (ALIGN). Running ARWEN with the “-ps < num>” option.

**Additional file 4: Table S4.** Annotation and gene organization of the mitochondrial genome of *C. floridensis*. N: minor strand; J: major strand. The tRNAs were annotated by different methods: MITOS2, ARWEN or aligning with the sequences of related species that were not annotated by software (ALIGN). Running ARWEN with the “-ps < num>” option.

**Additional file 5: Table S5.** Annotation and gene organization of the mitochondrial genome of *E. pela.* N: minor strand; J: major strand. The tRNAs were annotated by different methods: MITOS2, ARWEN or aligning with the sequences of related species that were not annotated by software (ALIGN). Running ARWEN with the “-ps < num>” option.

**Additional file 6: Table S6.** The Ks, Ka and Ka/Ks values in the mitogenomes of *S. coffeae*, *C. japonicus*, *C. rubens*, *C. floridensis* and *E. pela.* SC means *S. coffeae,* CJ means *C. japonicus,* CR means *C. rubens,* CF means *C. floridensis,* EP means *E. pela.* The base composition of the full genome was calculated with the major strand (+).

**Additional file 7:** **Table S7.** Codon usage of the 13 mitochondrial protein-coding genes of hemipteran insects. AA: amino acid; RSCU: relative synonymous codon usage.

**Additional file 8:** **Table S8.** Codon usage of the 13 mitochondrial protein-coding genes of five scale insects. AA: amino acid; RSCU: relative synonymous codon usage.

**Additional file 9: Fig. S1.** Circular map of mitogenomes of *S. coffeae*, *C. japonicus*, *C. rubens*, *C. floridensis* and *E. pela*. The genes on the outer loop were encoded on the major (J) strand, and others were on the minor (N) strand.

**Additional file 10: Fig. S2.** The secondary structures of the tRNAs in the *C. japonicus* mitogenome predicted by MITOS2 and ARWEN and drawn using RNAplot in ViennaRNAPackage 2.0. The tRNA genes are labelled with the amino acid abbreviations.

**Additional file 11: Fig. S3.** The secondary structures of the tRNAs in the *C. rubens* mitogenome predicted by MITOS2 and ARWEN and drawn using RNAplot in ViennaRNAPackage 2.0. The tRNA genes are labelled with the amino acid abbreviations.

**Additional file 12: Fig. S4.** The secondary structures of the tRNAs in the *C. floridensis* mitogenome predicted by MITOS2 and ARWEN and drawn using RNAplot in ViennaRNAPackage 2.0. The tRNA genes are labelled with the amino acid abbreviations.

**Additional file 13: Fig. S5.** The secondary structures of the tRNAs in the *E. pela* mitogenome predicted by MITOS2 and ARWEN and drawn using RNAplot in ViennaRNAPackage 2.0. The tRNA genes are labelled with the amino acid abbreviations.

## Data Availability

The dataset generated and/or analysed during the current study is available in the GenBank repository. The 488 accession numbers were obtained by conditional filtering (Hemiptera, mitochondrial, complete genome), and duplicate species were deleted. Direct link: https://www.ncbi.nlm.nih.gov/nuccore/?term=Hemiptera+mitochondrial+complete+genome.
