# Supplementary figures and images for: Mitochondrial genomes of soft scales (Hemiptera: Coccidae): features, structures and significance
Source: BMC Genomics. 2023 Jan 21;24:37. doi: 10.1186/s12864-023-09131-9 (PMC9863192; doi:10.1186/s12864-023-09131-9)

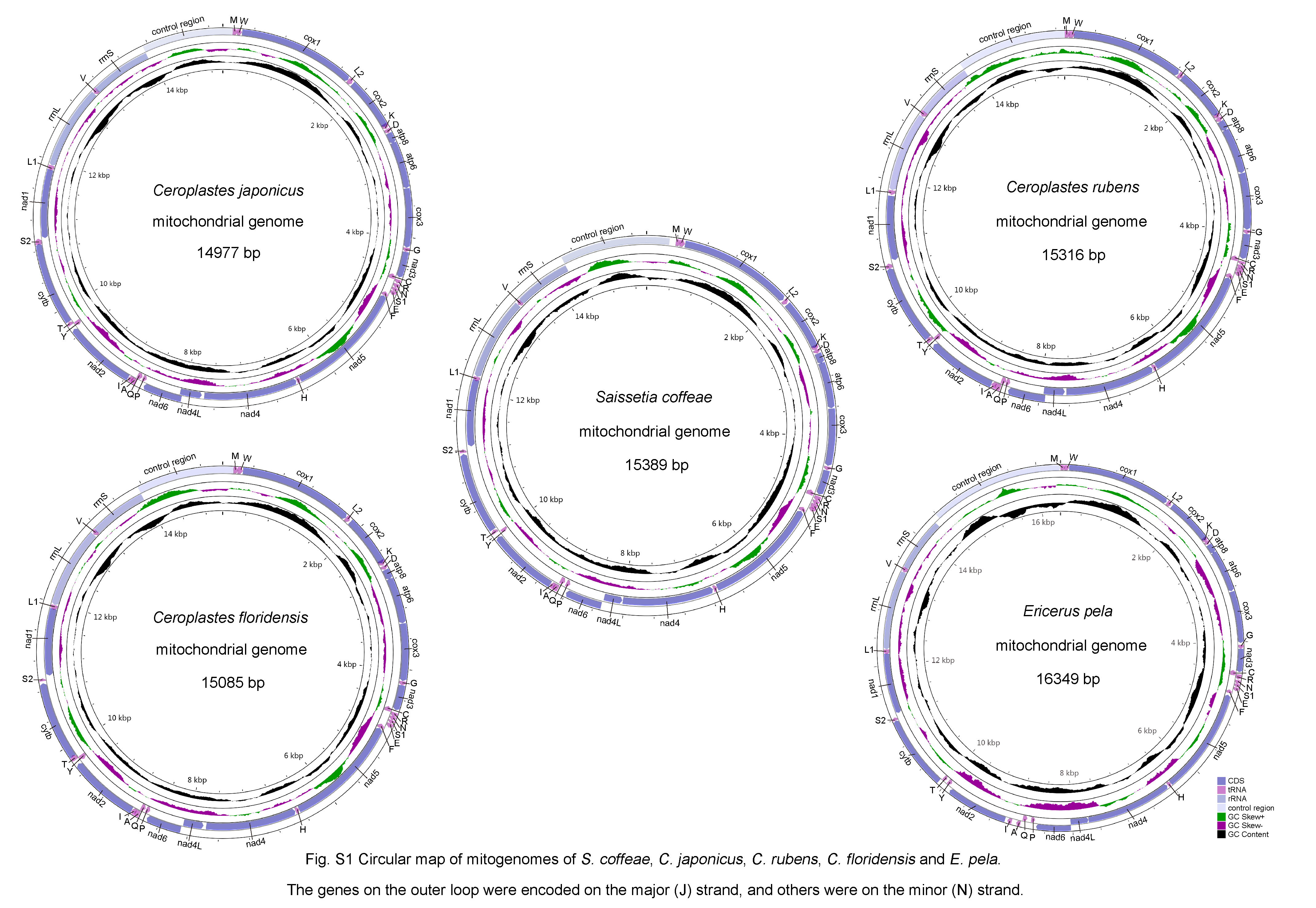

Supplement: Supplementary file 9 — Additional file 9: Fig. S1. Circular map of mitogenomes of S. coffeae, C. japonicus, C. rubens, C. floridensis and E. pela. The genes on the outer loop were encoded on the major (J) strand, and others were on the minor (N) strand. [file 12864_2023_9131_MOESM9_ESM.jpg]

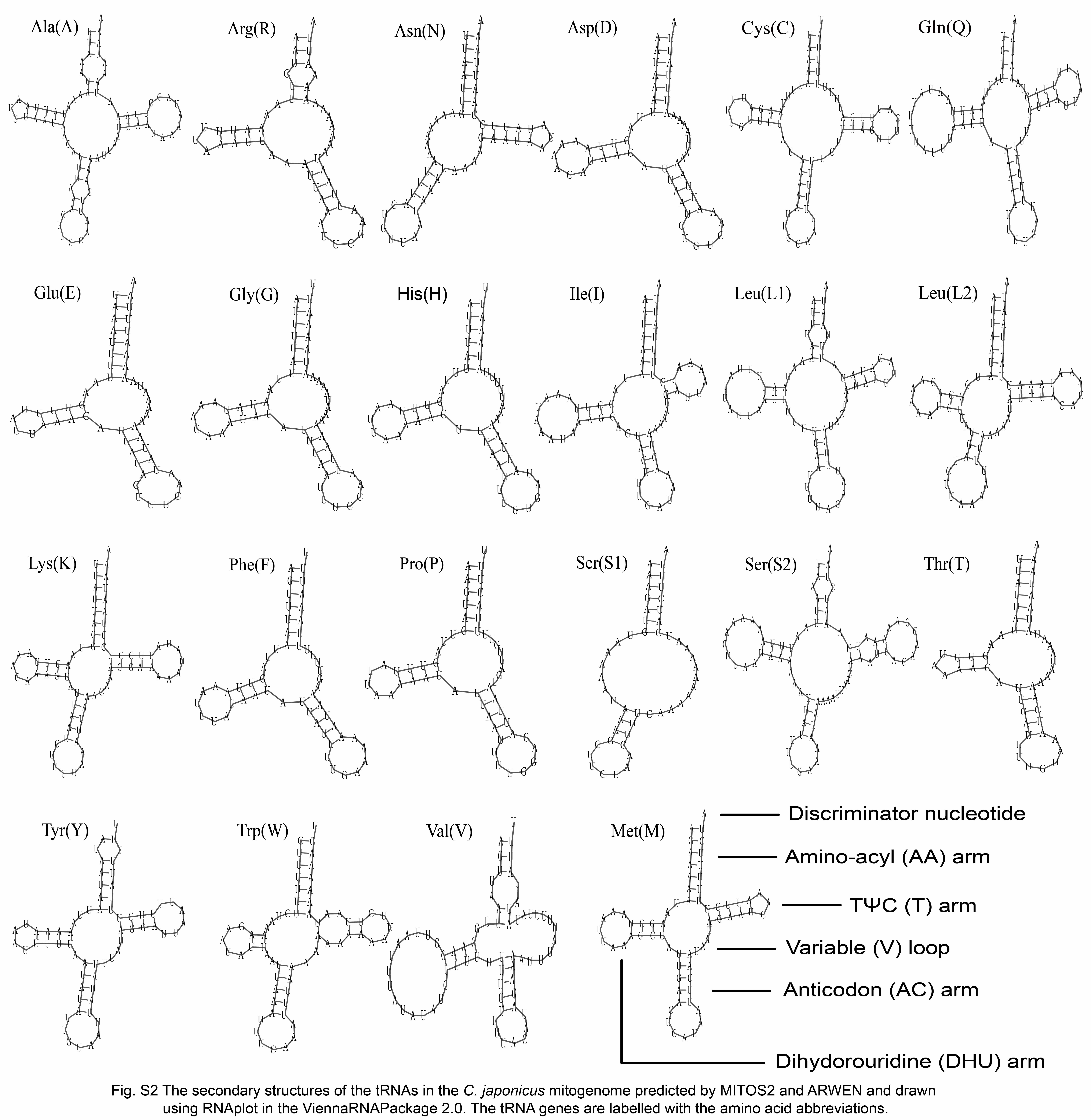

Supplement: Supplementary file 10 — Additional file 10: Fig. S2. The secondary structures of the tRNAs in the C. japonicus mitogenome predicted by MITOS2 and ARWEN and drawn using RNAplot in ViennaRNAPackage 2.0. The tRNA genes are labelled with the amino acid abbreviations. [file 12864_2023_9131_MOESM10_ESM.jpg]

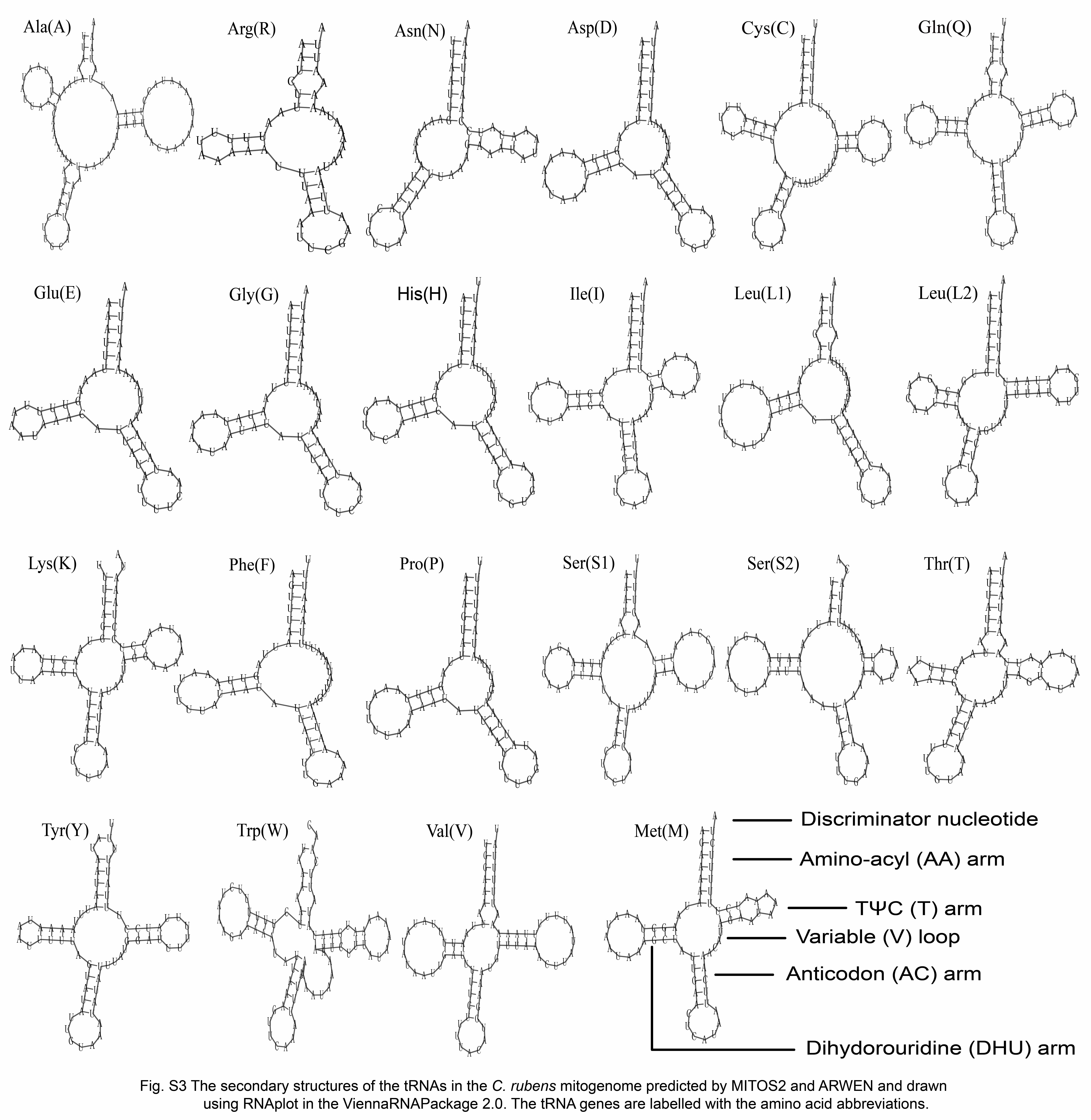

Supplement: Supplementary file 11 — Additional file 11: Fig. S3. The secondary structures of the tRNAs in the C. rubens mitogenome predicted by MITOS2 and ARWEN and drawn using RNAplot in ViennaRNAPackage 2.0. The tRNA genes are labelled with the amino acid abbreviations. [file 12864_2023_9131_MOESM11_ESM.jpg]

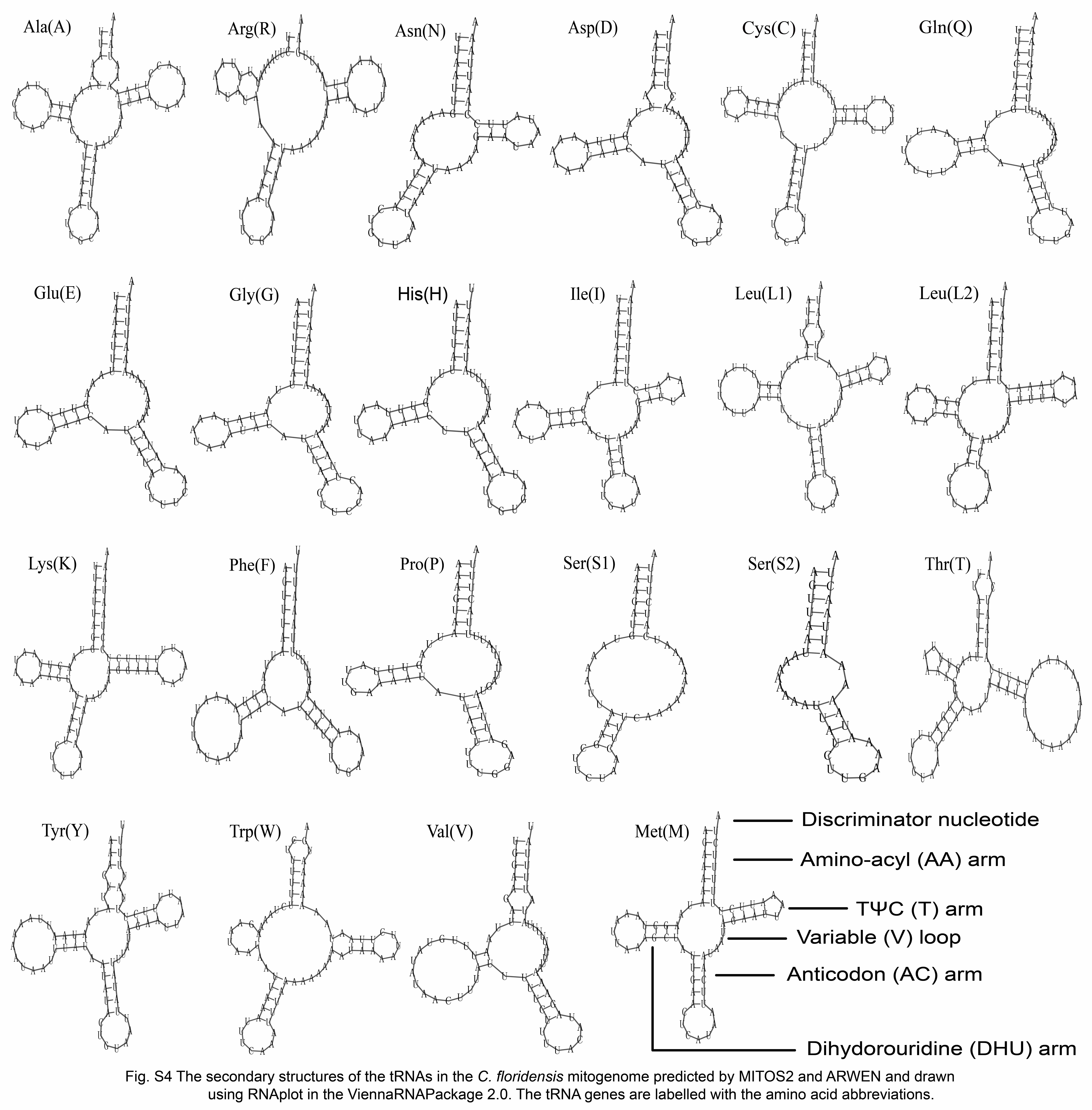

Supplement: Supplementary file 12 — Additional file 12: Fig. S4. The secondary structures of the tRNAs in the C. floridensis mitogenome predicted by MITOS2 and ARWEN and drawn using RNAplot in ViennaRNAPackage 2.0. The tRNA genes are labelled with the amino acid abbreviations. [file 12864_2023_9131_MOESM12_ESM.jpg]

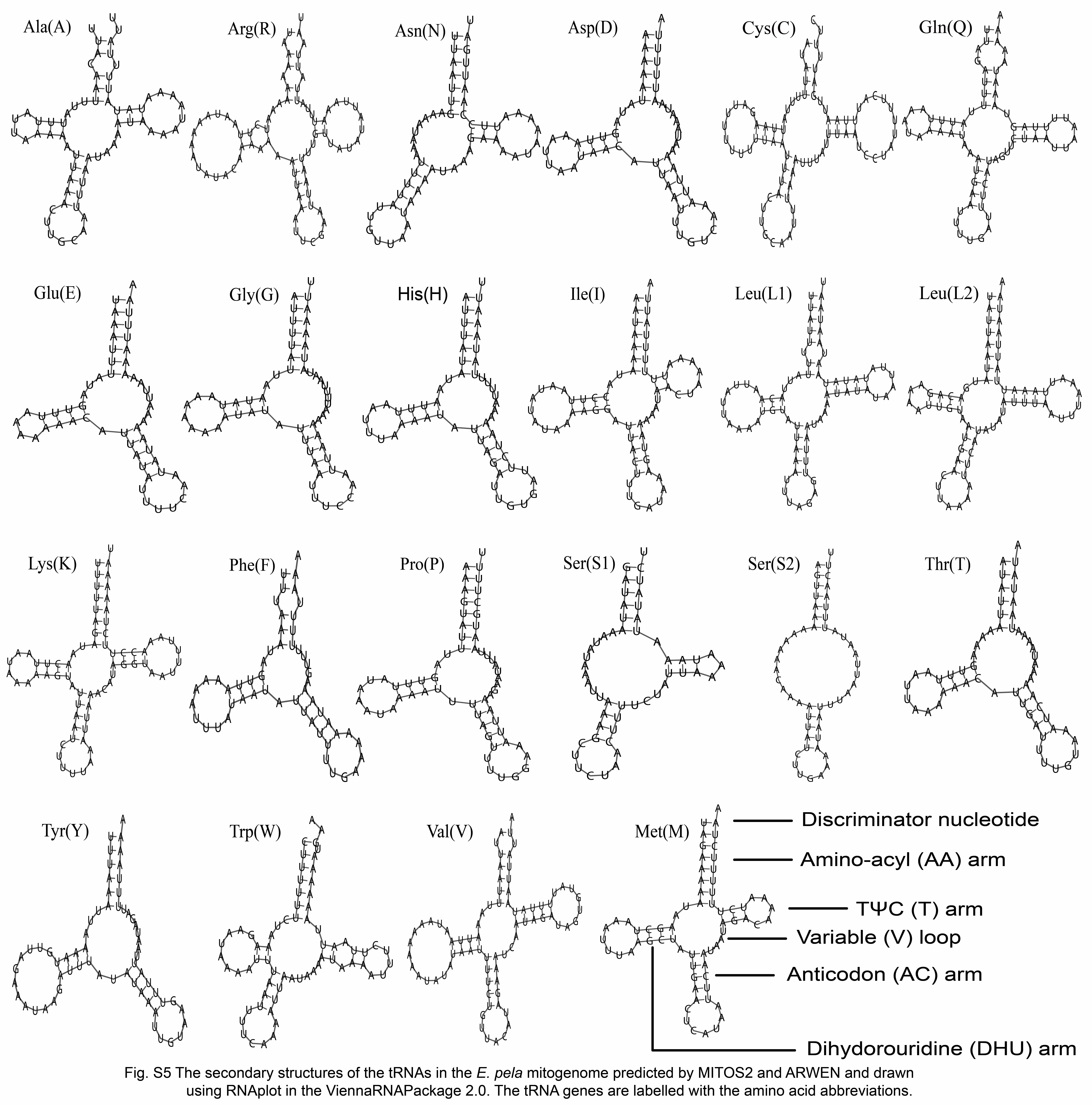

Supplement: Supplementary file 13 — Additional file 13: Fig. S5. The secondary structures of the tRNAs in the E. pela mitogenome predicted by MITOS2 and ARWEN and drawn using RNAplot in ViennaRNAPackage 2.0. The tRNA genes are labelled with the amino acid abbreviations. [file 12864_2023_9131_MOESM13_ESM.jpg]
